# Experimental annotation of post-translational features and translated coding regions in the pathogen *Salmonella *Typhimurium

**DOI:** 10.1186/1471-2164-12-433

**Published:** 2011-08-25

**Authors:** Charles Ansong, Nikola Tolić, Samuel O Purvine, Steffen Porwollik, Marcus Jones, Hyunjin Yoon, Samuel H Payne, Jessica L Martin, Meagan C Burnet, Matthew E Monroe, Pratap Venepally, Richard D Smith, Scott N Peterson, Fred Heffron, Michael McClelland, Joshua N Adkins

**Affiliations:** 1 Biological Sciences Division, Pacific Northwest National Laboratory, Richland, WA, 99352, USA; 2 Environmental Molecular Sciences Laboratory, Pacific Northwest National Laboratory, Richland, WA, 99352, USA; 3 Vaccine Research Institute of San Diego, San Diego, CA, 92121, USA; 4 Pathogen Functional Genomics Resource Center, J. Craig Venter Institute, Rockville, MD, 20850, USA; 5 Department of Molecular Microbiology and Immunology, Oregon Health and Sciences University, Portland, OR, 97239, USA

**Keywords:** gene annotation, proteomics, post-translational modifications

## Abstract

**Background:**

Complete and accurate genome annotation is crucial for comprehensive and systematic studies of biological systems. However, determining protein-coding genes for most new genomes is almost completely performed by inference using computational predictions with significant documented error rates (> 15%). Furthermore, gene prediction programs provide no information on biologically important post-translational processing events critical for protein function.

**Results:**

We experimentally annotated the bacterial pathogen *Salmonella *Typhimurium 14028, using "shotgun" proteomics to accurately uncover the translational landscape and post-translational features. The data provide protein-level experimental validation for approximately half of the predicted protein-coding genes in *Salmonella *and suggest revisions to several genes that appear to have incorrectly assigned translational start sites, including a potential novel alternate start codon. Additionally, we uncovered 12 non-annotated genes missed by gene prediction programs, as well as evidence suggesting a role for one of these novel ORFs in *Salmonella *pathogenesis. We also characterized post-translational features in the *Salmonella *genome, including chemical modifications and proteolytic cleavages. We find that bacteria have a much larger and more complex repertoire of chemical modifications than previously thought including several novel modifications. Our *in vivo *proteolysis data identified more than 130 signal peptide and N-terminal methionine cleavage events critical for protein function.

**Conclusion:**

This work highlights several ways in which application of proteomics data can improve the quality of genome annotations to facilitate novel biological insights and provides a comprehensive proteome map of *Salmonella *as a resource for systems analysis.

## Background

Many aspects of modern biological research are dependent on accurate identification of the protein-coding genes in each genome, as well as the nature of the mature functional protein products, a process commonly referred to as genome annotation. With the exponential increase in the number of sequenced prokaryotic genomes afforded by advances in genome sequencing technologies over the last decade, present day prokaryotic genome annotation is essentially an automated high-throughput process that relies heavily on *de novo *gene prediction programs [1-3].

While *de novo *gene prediction programs have significantly improved for prokaryotic genomes considerable challenges remain [4], such as determining the precise start and stop site of a gene, accurately predicting short genes, and determining a stop codon that represents an alternative amino acid rather than a true stop site. As efforts to sequence more branches of the tree of life expand, the level of accuracy for current gene prediction programs trained on proteobacteria datasets will markedly decrease, leading to an increase in incorrect predictions of protein-coding genes [4]. Compounding the issue is the lack of experimental evidence in support of predicted protein-coding regions for the overwhelming majority of annotated genomes. Where available, experimental evidence is typically based on expressed RNA sequences, such as from microarray or RNA Seq experiments. However, these genome-centric analyses do not independently and unequivocally determine whether a predicted protein-coding gene is translated into a protein or importantly provide any reliable information on post-translational processing.

Bottom-up proteomics offers the ability to directly measure peptides arising from expressed proteins representing the current best option for independently and unambiguously identifying at least an important subset of the protein-coding genes in a genome and can be used to experimentally validate gene annotations [4-9]. In a bottom up approach, proteins within a complex mixture are typically digested with a protease, after which the resulting peptides are separated by chromatographic methods and then analyzed using tandem mass spectrometry (MS/MS) [10,11]. Each MS/MS spectrum is a measure of fragment masses, ideally from a single peptide sequence of ~ 6-50 amino acids. This set of mass values is analogous to a 'fingerprint' that identifies the peptide. Interpretation of MS/MS peptide spectra is accomplished 1) by using algorithms such as X!Tandem [12], SEQUEST [13], or Mascot [14] to compare measured masses against a set of theoretical masses of possible protein sequences or 2) less commonly, by *de novo *analysis, which does not depend on any prior knowledge of the possible sequences [15,16]. Similar to searching MS/MS spectra against a set of predicted protein sequences, it is also possible (and feasible for simple genomes) to identify the protein-coding genes in a genome by searching MS/MS spectra against a six-frame translation of the genomic DNA sequence, thereby precluding the inherent biases derived from gene prediction methods. We note that scaling up to an exponentially larger database, as a result of six-frame translation of a genomic DNA sequence, makes searches slower by potentially orders of magnitude. Additionally, it also results in increased possibility for false-positive identifications as the false discovery rate scales with the increasing database size, greatly decreasing sensitivity; twin challenges that can only be feasibly met with dedicated/sophisticated computing resources precluding routine use at present.

Bottom-up proteomics also provides an avenue for obtaining biologically relevant information about post-translational modifications. Even for relatively simple biological systems such as prokaryotes, post-translational modification (PTM) events are increasingly being recognized as important, but are poorly characterized with regard to sites or function. While PTM information gained from genome-scale MS/MS datasets stands to benefit biological understanding of bacterial organisms, significant technological challenges hamper the routine inclusion of this valuable information in primary annotations. This situation is exemplified by only a single report of genome-scale MS/MS datasets being used to comprehensively annotate post-translational modifications events in a genome sequence [17]. However, the low resolution of the MS/MS datasets makes assignment of modifications, including crucially the site of modification, less confident and ill suited for routine use. For example, the 0.036 Dalton mass difference between Gln (Q) and Lys (K) cannot be resolved in low resolution MS/MS spectra, which increases the number of possible peptide candidates to be matched and reduces confidence in assignments. On the other hand, the sequencing precision afforded by high resolution MS/MS spectra can resolve residues with small mass differences like Gln and Lys, which reduces the search space and thus the computational burden, and increases confidence in assignments.

In this study, we employed a bottom-up proteomics approach supplemented with a recently described *de novo *sequencing methodology using high resolution and high mass measurement accuracy MS/MS data [18,19] to accurately uncover the translational landscape and post-translational features of the bacterial pathogen *Salmonella enterica *serovar Typhimurium (STM) 14028. *Salmonella *Typhimurium is a leading cause of bacterial gastroenteritis and is widely used as a model to investigate basic genetic mechanisms as well as the interaction between bacterial pathogens and mammalian hosts. In spite of the clinical and basic science relevance of *Salmonella *Typhimurium there has been no comprehensive analysis undertaken to provide experimental support of its *in silico*-based genome annotation to facilitate systems-level analysis. Our data provides protein-level experimental validation for approximately half of the predicted protein-coding genes in STM 14028 and suggests revisions to 47 genes assigned incorrect translational start sites, including a potential novel alternate start codon. Additionally, we uncovered 12 non-annotated genes missed by gene prediction programs, as well as evidence suggesting a role for one of these novel ORFs in *Salmonella *pathogenesis. We also characterized post-translational features in the STM 14028 genome, including chemical modifications and proteolytic cleavages. We find that bacteria have a much larger and complex repertoire of chemical modifications than previously thought including several novel modifications. Our *in vivo *proteolysis data identified more than 130 signal peptide and N-terminal methionine cleavage events critical for protein function.

Contrary to the overwhelming majority of proteogenomics analyses that utilize proteomics data to improve the quality and completeness of previously annotated genomes, this study represents one of the first to utilize proteomics data "as part of a largely automated, high-throughput annotation process directly at the primary stage of genome annotation" [20].

## Results and discussion

The genome of *Salmonella *Typhimurium (STM) 14028s was sequenced as described in the Methods section and is composed of two replicons: a main chromosome (4.87 Mb) and a plasmid (94 kb) with over 99% sequence homology to the *Salmonella *Typhimurium LT2 virulence plasmid pSLT. The automated annotation pipeline at the J. Craig Venter Institute (JCVI) was employed to identify genome features in the raw DNA sequence, gather evidence for function of the features, and assign functional annotation. The main genome characteristics of STM 14028 are presented in Table 1, and the full list of predicted protein-coding genes is provided in Additional file 1 Table S1. For the proteogenomic analysis described in this study we focused on chromosome-encoded genome features. Low resolution liquid chromatography (LC)-MS/MS datasets were employed to experimentally validate gene annotations and high resolution LC-MS/MS datasets were employed to accurately annotate post-translational features as described below.

**Table 1:** General features of *S*. Typhimurium 14028 genome

Parameter	Chromosome	Plasmid
Size (bp)	4.87 Mb	94 kb
G+C content	52.2%	53.1%
rRNA	16	0
tRNA	63	0
Protein-coding genes	4816	126

To confirm and correct STM 14028 annotations and in particular to identify proteins specified by non-annotated genes, we searched low resolution MS/MS spectra from 330 individual LC-MS/MS analyses against a six-frame translation of the STM 14028 genome sequence. These data comprise STM 14028 samples from different cell culture conditions designed to mimic various aspects of the non-infectious and infectious environments experienced by *Salmonella *Typhimurium. Briefly, these included Luria-Bertani (LB) logarithmic and LB stationary phases, and two acidic minimal medium (AMM) conditions, AMM1 and AMM2 [21,22]. The mass spectrometry datasets employed in this proteogenomic annotation study have been previously described, detailing the annotated *Salmonella *Typhimurium proteome response to the above growth conditions [21,23,24]. Peptides with either one or two tryptic ends were filtered according to the parameters described in the Methods section. As an additional confidence metric we only retained those filter-passing peptides that mapped to unique locations (i.e. a unique ORF) in the six-frame translation. Peptides mapping to multiple locations (i.e. different ORFs) in the six-frame translation introduce a potential source of ambiguity making it difficult to confidently validate individual ORFs or correct annotation errors. A total of 23889 unique peptides that passed the above criteria were identified with a false discovery rate of < 1%. Of these, 23097 peptides were assigned as being generated by two tryptic ends and 791, with one tryptic end.

### Experimental validation of predicted genes at the protein level

For the majority of genes annotated as part of sequencing efforts there is no direct experimental evidence that the gene is translated into a protein. In cases where experimental evidence is available it is typically based on expressed RNA sequences, such as from microarray or RNASeq experiments. It is clear however that expressed RNA sequences cannot independently and unequivocally determine whether a predicted protein-coding gene is translated into a protein. Thus knowing that a gene or part of it in the relevant frame is being made (i.e. translated into protein) is useful information.

**Figure 1:**
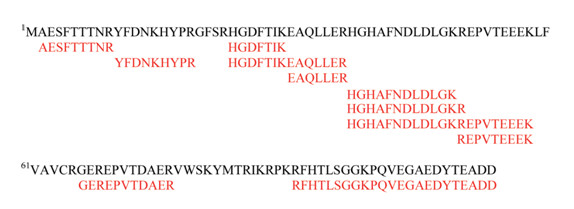
**Validation of computationally predicted genes**. Multiple peptides mapping to predicted gene ORF04105 evidence for the expression of the product(s) encoded by ORF04105. Protein sequence of ORF04105 shown in black, and the identified peptides are shown in red.

Multiple unique peptides mapping to a single gene provide compelling evidence for the expression of the product(s) encoded by that particular gene. As such, we matched the 23,889 identified unique peptides to the 4817 predicted chromosomal genes in the STM 14028 genome sequence to validate automated gene predictions at the protein level. The predicted genes covered most of the observed peptides (23,759 of the 23,889). Conservatively, a gene product was confirmed expressed at the protein level only when a minimum of two unique peptides mapped to the particular gene. Using this criterion, our proteomics data validated 2118 of the 4817 annotated chromosomal ORFs in the STM14028 genome sequence at the protein level (Additional file 1 Table S2), providing experimental evidence for the products of these genes. Figure 1 shows an example of a predicted gene whose length is covered by multiple identified peptides providing experimental evidence for its expression at the protein level. Given the limited number of possible tryptic peptides available for protein identification in small ORFs, the above criteria would presumably preclude the confident identification of novel small ORFs.

The ~44% level of proteome coverage is in line with, and in most published cases exceeds, results obtained in both typical proteomics experiments [25-27] and previous proteogenomic analyses [17,28,29]. Typical coverage is in the 30% range for the annotated ORFs. However, multiple factors potentially prevent 100% coverage, including extensive post-transcriptional regulation that makes transcripts rather poor guides for protein expression [21,30]. Here we have used four differing growth conditions including typical laboratory conditions and an infection-like environment. However, not all possible growth conditions for *Salmonella *Typhimurium can be efficiently sampled and it is likely that certain ORFs require specific conditions for expression, such as intracellular growth in host cells [31]. In addition, various technical aspects associated with our experiments may have precluded detection, such as incompatibility with buffers for soluble protein extraction. The two peptide criterion may also negatively impact coverage of small proteins present in low concentrations. However it is possible that application of additional protein and peptide separation methods could enable improved coverage of low abundance proteins.

### Refinement of predicted gene structures: Start sites

Determining the correct start position of a gene remains a challenge for current gene prediction algorithms. In a recent re-analysis of 143 annotated prokaryotic genomes, Nielsen and Krogh [32] observed that in some genomes up to 60% of the genes may have been annotated with a wrong start codon, especially in GC-rich genomes. Accurate start site predictions better define intergenic spaces that may encode promoters and regulatory binding sites, which are critical elements in studies of transcriptional regulation. Cellular localization signals also are contained in start sites, which makes accurate start site predictions important for accurately determining the localization of proteins within a cell.

Peptides that map to genomic regions within 200 bp upstream of previously annotated genes and in the same translational reading frame represent evidence supporting extensions of their predicted start sites. We note that this approach has been described in detail in a number of recent publications [17,29,33]. Additionally peptides that span (i.e., partially overlap) the start site of previously annotated genes also represent evidence supporting extensions of their predicted start sites. To this end, we examined the identified peptides for experimental evidence supporting extension of start sites in each of the predicted genes, and where possible proposed a new start codon. 51 peptides spanned the start site of previously annotated genes, and 36 peptides mapped to genomic regions within 200bp upstream of previously annotated genes. Overall, 87 of the 23,889 peptides mapped to genomic regions that served as experimental evidence supporting extension of start sites. We restricted our correction of start sites to predicted genes confirmed at the protein level (see preceding section), which yielded a final candidate list of 75 peptides that correspond to 47 genes requiring N-terminal extension/start site correction (Additional file 1 Table S3A). New start sites were largely defined by the first Methionine amino acid and/or start codon encountered upstream of peptides mapped to the genomic region within 200 bp upstream of their previously annotated start sites.

**Figure 2:**
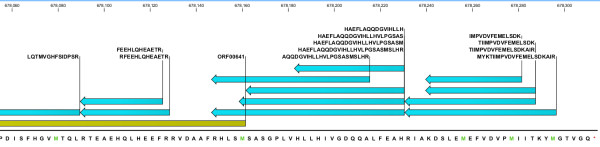
**Correcting start site assignment for ORF00641**. Multiple peptides (green bars) map upstream of the predicted ORF00641 (yellow bar) including several peptides that spans the currently predicted gene start, providing evidence in support of extending the start site. The in-frame * symbol represents the stop codon TAA.

**Figure 3:**
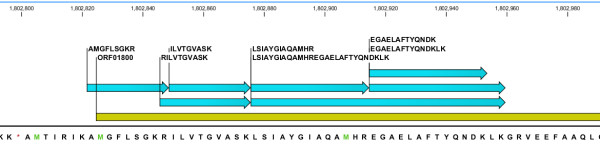
**Correcting start site assignment for ORF01800**. Multiple peptides (green bars) map to predicted ORF01800 (yellow bar) including one peptide that spans the currently predicted gene start, providing evidence in support of extending the start site. The in-frame * symbol represents the stop codon TAA.

Figures 2 and 3 highlight examples depicting the utility of proteomics data for start site correction. ORF0641 is a member of the universal stress protein (Usp) family of proteins with biological roles in motility, adhesion and coping with stress [34]. In our proteomics data we observed several peptides upstream of ORF00641 in the same reading frame and within 200 bp of the predicted start site, i.e., evidence supporting extension of the predicted start site (Figure 2). Furthermore, three of these peptides span the previously predicted start site, providing additional evidence in support of refining the start site. ORF01800 is the NADH-dependent enoyl reductase from the type II bacterial fatty acid biosynthesis pathway (FAS-II), as inferred from the primary *in silico *genome annotation, a validated but currently underexploited target for drug discovery [35]. Similarly, we observed several peptides that overlap this ORF including one peptide that spanned the currently predicted gene start (Figure 3). The accurate experimental delineation of the structure of this clinically relevant gene represents an important step in facilitating ongoing drug development efforts. While beyond the scope of this study, we remark that protein expression and purification coupled with Edman degradation represents a useful orthogonal avenue to validate at the protein level the corrected assignments proposed.

**Figure 4:**
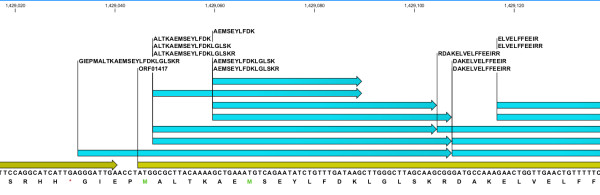
**Evidence for potential novel alternative start codon**. Multiple peptides (green bars) map to predicted ORF01417 (yellow bar) including one peptide that spans the currently predicted gene start, providing evidence in support of extending the start site. The in-frame * symbol represents the stop codon TGA.

**Figure 5:**
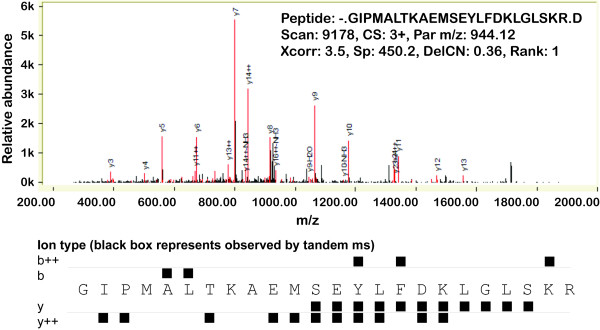
**Validation of mass spectral evidence**. Annotated experimental MS/MS spectra of the peptide "GIEPMALTKAEMSEYLFDKLGLSKR" with the fragmentation ladder below.

In prokaryotes the initiation of translation is typically mediated by the start codon ATG. In addition GTG and TTG are also used as alternative start codon. Studies in *E. coli *have estimated the frequency of start codon usage as ATG 83%, GTG 14% and TTG 3%. However a very small number of studies have also reported CTG, ATT, ATA, and ATC codons to function as rare non-canonical translation initiators. Interestingly our proteomics data showed multiple peptides that overlap ORF01417 (integration host factor alpha) including one peptide that spanned the currently predicted gene start, providing evidence in support of extending the start site (Figure 4). The peptide, GIEPMALTKAEMSEYLFDKLGLSKR, is immediately adjacent to a stop codon (TGA) with another stop codon (TAA) a further seven amino acids upstream making the possibility that the peptide results from a stop codon read through unlikely. Considering this, the first amino acid in the peptide sequence Glycine (G) would presumably be the translation initiator. Glycine is coded for by the triplet codon GGG. However, it is well known that even if an alternative start codon is used as the translation initiator, it gets translated as methionine. Thus we examined the possibility of a frame-shift involving the preceding ORF pheT, coded in a different frame, as a potential mechanism to explain this observation. However, the ORF pheT terminates with the "...ASLRD-stop" amino acid sequence conserved across several related *Salmonella *and *E. coli *strains. It is clear that further studies beyond the scope of the present work will be required to resolve and or confirm this interesting observation. The peptide GIEPMALTKAEMSEYLFDKLGLSKR is defined as fully tryptic, having a native N-terminus and a C-terminal arginine, and was observed in three independent LC-MS/MS analyses with minimum SEQUEST^® ^peptide identification scores across the three independent LC-MS/MS analyses of Xcorr ≥ 3.47, ΔCN ≥ 0.23, and PeptideProphet™ probability ≥ 0.99. A representative MS/MS spectrum of the peptide GIEPMALTKAEMSEYLFDKLGLSKR is shown in Figure 5. While we do not observe any fragments ions corresponding to proline directed fragmentation, the near complete y ion series and extensive sequence coverage when considering both b and y ion series provides additional confidence to the annotation.

### Identification of novel genes

The identification of protein-coding genes (structural annotation) in eukaryotic genomes is complicated by the high frequency of alternative splicing in most eukaryotic genes. Additionally the small fraction of protein-coding genes that comprise eukaryotic genomes (< 25% in worms and < 5% in humans) make the identification of coding sequences against the ubiquitous background of non-coding sequences difficult [36]. In contrast, the usual absence of introns and the compact nature of prokaryotic genomes make the identification of all possible ORFs longer than a chosen threshold in a DNA sequence a relatively straightforward computational exercise. This view, in particular of bacterial genomes, has lead to the assumption that computationally derived coding sequences completely describe the entire coding capacity of a bacterial genome. However, structural annotation of prokaryotic genome sequences by predicting coding sequences is far from being a trivial matter. A number of recent studies [7,17,37] used proteomics data to identify novel protein-coding genes in prokaryotic genomes that had been missed by *de-novo *gene finding programs.

Peptides that map to genomic regions outside the boundaries of predicted genes are evidence suggestive of the presence of novel genes missed by gene finding programs. By matching the 23,889 identified peptides to the 4817 predicted chromosomal genes in the STM 14028 genome sequence, we detected 130 peptides mapped to regions falling outside the boundaries of known protein coding genes. We further refined the list of peptides by excluding the 36 peptides that mapped to genomic regions within 200 bp upstream of predicted genes, i.e., peptides that are indicative of N-terminal extensions (see previous section), which yielded a final candidate list of 92 peptides (Additional file 1 Table S4A). These peptides represent experimental evidence for the presence of novel genes missed by gene finding programs. Using the set of 92 intergenic peptides, we defined 12 novel genes that had been missed by gene prediction methods. Note that novel genes were confirmed as detected and expressed at the protein level only when a minimum of two peptides mapped to that particular novel gene.

**Figure 6:**
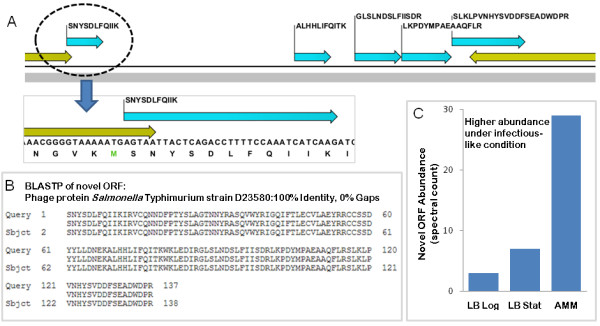
**Identification of novel genes**. A) Five proteomics identified peptides map to the genomic region 795142 to 795556 on the forward strand, where no gene had been previously predicted by computational approaches. B) Sequence alignment shows 100% homology to a phage protein in *Salmonella *Typhimurium strain D23580. C) Examination of protein expression shows the novel ORF is highly expressed under infection-mimicking conditions.

Of particular interest was the novel ORF C1368_1:795109-795576 defined by five peptides that map to the genomic region 795142 to 795556 where no gene had been predicted (Figure 6A). Protein BLAST analysis (BLASTP) of the genomic region where the five peptides mapped to against the non-redundant protein sequences database revealed a single significant hit to a phage protein in *Salmonella *Typhimurium strain D23580 (Figure 6B). Interestingly, examination of our experimental MS/MS data showed that this novel ORF C1368_1:795109-795576 was highly expressed under growth in acidic minimal media (AMM) relative to growth under standard laboratory conditions (Figure 6C). Growth in AMM has been shown to approximate the environment found within the *Salmonella*-containing vacuoles (SCV) observed in infected host macrophages, and we among others have shown that this media induces the expression of the *Salmonella *pathogenicity island 2 (SPI-2)-encoded type-III secretion system (TTSS), a system critical for virulence and intramacrophage survival [21,22,38,39]. Thus proteins co-regulated with known virulence proteins under such environmental conditions are likely to play a role in *Salmonella *Typhimurium pathogenesis. Indeed phage proteins are known to play an important role in *Salmonella *Typhimurium pathogenesis further supporting our observation [40]. The full repertoire of factors employed by *Salmonella *Typhimurium to establish a successful infection is still unknown, presumably because they remain undetected due to a number of reasons including being missed by annotators; thus the enumeration of all proteins in the *Salmonella *Typhimurium 14028 genome, especially those co-regulated with known virulence factors, as presented above is an important step in understanding the mechanisms of *Salmonella *Typhimurium pathogenesis.

### Assessing the performance of proteogenomic annotation

Manual curation has become a luxury reserved for a few organisms and the majority of newly sequenced genomes only receive a single round of computational annotation with no additional manual refinement, therefore the utility of proteogenomics lies in its application at the primary stage of genome annotation to improve the quality and completeness of the automated genome annotation. A version of the STM14028 genome annotated using a combination of computational methods and human annotators, i.e. refined annotation, released while this manuscript was in preparation [41] allowed us to assess the performance of proteogenomic annotation.

A comparison of the new start sites for the 47 genes proposed by the proteomics data to the start sites for the same 47 genes in the refined STM14028 genome annotation revealed an overlap of 34 genes where start sites matched exactly (Additional file 1 Table S3B). Of the remaining 13 genes, 10 had proteomics-suggested start sites that were downstream of those determined by refined annotation. While the proteomics data suggest a shorter ORF than indicated in the refined annotation, homology analysis (BLASTP) supports the refined annotation. Note that deeper proteome coverage may improve matches to the refined annotation. The remaining three genes had proteomics-suggested start sites that were upstream of start sites in the refined annotation, which is tangible experimental evidence that suggests a longer ORF than indicated in the refined annotation, i.e., correcting the refined annotation. In summary 44 of 47 start site corrections proposed by our proteogenomic analysis in the primary annotation reported in this study were in line with the refined annotation. The remaining three start site corrections suggested represent corrections to both the automated (this study) and refined annotation [41].

Our proteogenomic analysis also identified 12 novel genes missed by gene prediction methods. A comparison of the 12 novel genes proposed by proteomics data to the refined STM14028 genome annotation revealed 9 of the 12 novel genes were also identified and annotated similarly in the refined annotation (Additional file 1 Table S4B). This result highlights the power of proteomics data to identify relevant novel protein-coding genes. The three remaining proteomics-identified genes missed by *de novo *gene finding programs and human annotators represent a correction and improvement to both the current automated (this study) and refined [41] annotations.

### Annotation of complex post-translational chemical modifications

While a number of groups have used genome-scale MS/MS data to confirm predicted bacterial genes at the protein level, as well as identify new genes and correct gene prediction [37,42-44], there is only a single report of genome-scale MS/MS data being used for comprehensive "unrestricted" analysis and annotation of post-translational chemical modifications (PTCMs) in a bacterial system [17]. We note however a number of recent studies focused on characterizing ribosomal protein modifications [45,46]. As relatively little is known about PTCMs in bacteria, even for intensively studied model organisms such as *E. coli *and *Salmonella*, any PTCM information gained from genome-scale MS/MS data would aid biological understanding of bacterial organisms.

We have recently described a *de novo *sequencing approach (*de novo*-UStags), using high resolution and high mass measurement accuracy MS/MS data, for the accurate discovery of unknown or unexpected PTCMs of proteins [18,19]. Here we apply the *de novo*-UStags approach to analyze 60 high resolution LC-MS/MS datasets for PTCMs in the STM 14028 genome. These datasets represented samples that had been grown in a variety of cell culture conditions.

**Figure 7:**
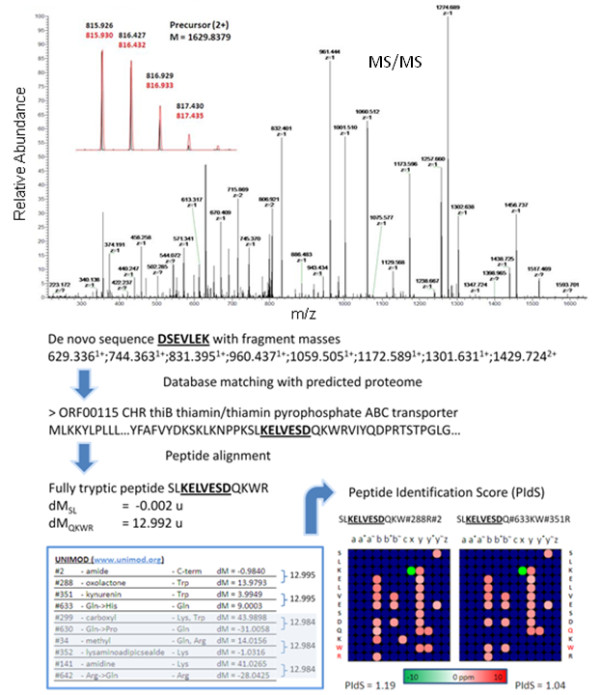
**Post-translational chemical medication (PTCM) analysis using *de novo*-Ustag approach**. An example illustrating the application of *de novo*-UStag approach in detection of multiple modification and high resolution MS/MS for distinction between multiple possible combinations. C-terminus amidation and tryptophan oxidation to oxolactone was determined for the sequence SLKELVESDQKWR of the Salmonella thiamin/thiamin pyrophosphate ABC transporter protein. Explanation for the determination of modifications is detailed in text.

Figure 7 illustrates application of the *de novo*-UStags approach in conjunction with the UNIMOD database to reveal multiple PTCMs, on a single sequence. The UStag DSEVLEK was sequenced from deisotoped high resolution MS/MS spectra and matching with the predicted proteome located this UStag to residues Lys142-Asp148 of the *Salmonella *Typhimurium protein thiamin/thiamin pyrophosphate ABC transporter. The measured mass of the UStag suffix sequence and the predicted mass have an apparent mass difference (dm) of -0.002 atomic mass units (u), revealing no modification on the suffix. The prefix had an apparent dm of 12.992 u from the database predicted sequence, a discrepancy that suggests a peptide modification. The mass shift of 12.992 u can be explained by several combinations of 2 modifications in various arrangements on the prefix sequence QKWR within a 10 parts per million (ppm) mass tolerance according to UNIMOD database as shown in Figure 7. Using an in-house scoring function (described in Additional file 2) that takes into account sequencing precision and count of isotopically resolved b and y fragments among other parameters, the 12.992 u mass shift is best explained by a C-terminus amidation combined with tryptophan oxidation to oxolactone.

Using the approach illustrated above, we determined mass shifts for UStags in 4144 MS/MS spectra that represented 675 proteins. We estimate the false discovery rate (FDR) of PTCM analysis to be < 1% (see Methods). Utilizing in-house developed software and a list of 450 UNIMOD modifications, including SNP substitutions, http://www.unimod.org (Additional file 3 Table S5F) we inferred hypothetical explanations for observed mass shifts in ~92% of the spectra in which modifications were detected (i.e. 3826 MS/MS spectra), several of which had multiple potential chemical explanations (Additional file 3 Table S5D). Where applicable observation of "differential" fragments were used to resolve ambiguity of assignments between SNPs and other modifications of similar mass defect as described below and in the Methods and Additional file 2. The remaining spectra (~8%, i.e. 318 MS/MS spectra) in which modifications were detected but where no plausible modification combination of up to two of the selected 450 UNIMOD modifications could explain observed mass shift for prefix or suffix sequence represent novel previously un-described PTCMs and are listed in Additional file 3 Table S5E. To obtain lower limit estimates on the number of distinct PTCMs, we binned different mass shifts and inferred a total of 70 distinct modifications mass-shifts, each with multiple potential chemical explanations according to UNIMOD (Additional file 3 Table S5A). This represents a much larger and complex repertoire of chemical modifications than previously thought existed in bacteria. Note that this only considered mass-shifts for which a potential chemical explanation was found in UNIMOD, thus most likely an underestimate. Nevertheless this estimate represents a much larger number of modification types than can be considered by commonly used, but often restrictive PTCM search algorithms such as Sequest, X!Tandem, and Mascot [12-14]. The 3826 modification containing MS/MS spectra with hypothetical explanations for the observed mass shifts were ranked using a peptide identification scoring function (see Methods and Additional file 2) and based on the identification scores we obtained unambiguous explanations, with regards to type, number and site of modification, for observed mass shifts in 1273 MS/MS spectra (i.e., ~31% of modification containing spectra, see Additional file 3 Table S5B). We also established confident assignment of number and type(s) of modification to explain observed mass shifts in an additional 239 MS/MS spectra (i.e., ~6% of modification-containing spectra); however site(s) of modification could not be unambiguously derived from spectral evidence (Additional file 3 Table S5C).

Among the modification types observed were those known to result from sample preparation, including carbamylation and carbamidomethyl, as well as those that can occur both *in vitro *and *in vivo*, such as methionine oxidation [47,48] and asparagine deamidation [49-51]. As no methods are currently available for distinguishing between *in vitro *and *in vivo *modifications, to identify PTCMs in STM that are biologically relevant we assumed such modifications would be conserved across closely related organisms. Using this approach we report a number of PTCMs of biological significance previously unappreciated in STM. Methylation of ribosomal proteins has been suggested to modulate the intra- or intermolecular interactions of the methylated ribosomal proteins or affects their affinity for RNA, and, thus, influences various cell processes, including ribosome assembly and translation accuracy [52,53]. Single methylation of the *E. coli *ribosomal protein L7/12 has been reported [54] and localized to K82 [55], while the ribosomal protein L3 has been reported as being methylated at Q150 in *E. coli *[56]. In the present study we observe methylation of the *E. coli *ribosomal protein L7/12 homolog in STM14028 ORF04348 at the same location, K82, and methylation of the ribosomal protein L3 ortholog in STM14028 ORF03631 at the exact same position, Q150, suggesting a similar regulatory and/or structural role in STM14028 as in *E. coli *(Additional file 4 Figure S1; 3 methylated residues shown in red). Methylation of the translation elongation factor Tu (tufB) at position K56 has been reported, and suggested as a mechanism for 'fine tuning' of tufB-tRNA inter-molecular interactions [57]. We observed the same modification at K57 of the STM14028 protein ORF03636, the homologous position to K56 of the *E. coli *protein, suggesting a similar functional role in STM14028 as in *E. coli *(Additional file 4 Figure S1; modified residue shown in red).

**Figure 8:**
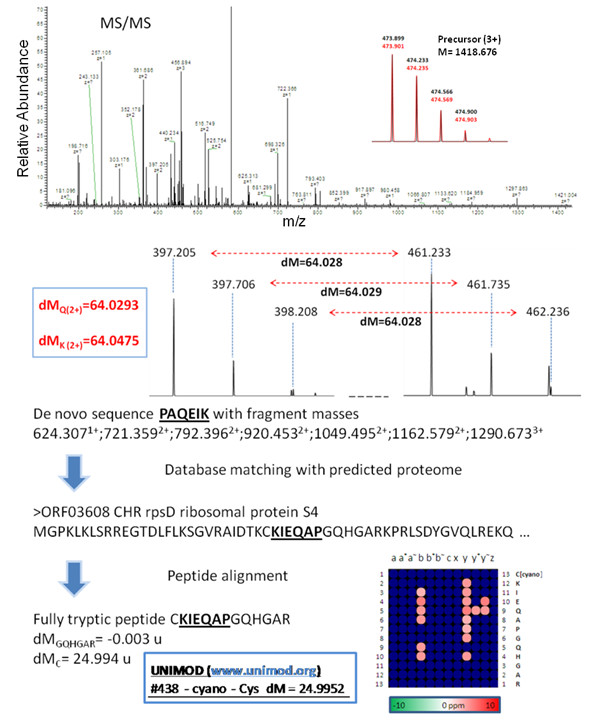
**Identification of a novel PTCM in *Salmonella***. Cyano modification on Cys was determined for the sequence CKIEQAPGQHGAR of the *Salmonella *ribosomal protein S4. Explanation for the determination of modifications is detailed in text. Inset figure demonstrates how sequencing precision in high resolution MS/MS spectra easily resolves residues with close mass like Gln (Q) and Lys (K) which cannot be resolved in low resolution spectra. The map in the lower right corner of contains all predicted fragments observed from the resolved isotopic clusters with shade of spots corresponding to parts per million (ppm) distance from expected value. This map is used to determine the correct identification between all plausible sequences and PTCMs matching observed mass shifts.

Besides those PTCMs with functional inferences from comparative analysis we observed additional *Salmonella *Typhimurium PTCMs of interest. For example, Figure 8 shows the detection of a cyano modification at residue Cys28 of the *Salmonella *Typhimurium protein ribosomal protein S4 (RpsD). Note that the cyano modification is classified in UNIMOD as a post-translational modification and not a chemical derivative/artifact. This to our knowledge is the first report of an *in vivo *cyano modification in *Salmonella*. Previous studies in *E. coli *have reported on the effects of cyanate on the different catalytic activities of carbamyl phosphate synthetase [58,59]. It will be interesting to assess the biological significance of cyano modification on RpsD. While the current lack of available experimental data with regard to PTCMs for *Salmonella *Typhimurium (and bacteria as a whole) prohibits us from validating predicted modifications presented herein, future *Salmonella *Typhimurium studies may confirm several of these putative modifications, leading to a greater understanding of the biology of this pathogenic organism.

### Annotation of post-translational proteolytic events

Proteolytic cleavage plays an essential role in the control of numerous biological processes, including protein localization, fate and activity as well as the processing of cellular information. However current high throughput genome annotation pipelines are blind to this information, any amount of which would clearly improve the quality of the genome annotation. The low throughput and labor-intensive nature of Edman degradation and two dimensional gel electrophoresis approaches make them incompatible with state of the art high throughput annotation pipelines. Using the high precision MS/MS data generated above, we highlight the use of proteomics data to identify a subset of post-translational proteolytic events in a high throughput label-free manner and include this additional layer of information to improve the quality of the genome annotation.

Data were searched against the predicted genes in the STM 14028 genome sequence using X!Tandem [12] and included 10336 unique fully tryptic peptides (i.e., two inferred tryptic ends), 1756 with one tryptic end (partially-tryptic), and 20 with no tryptic ends (non-tryptic), identified at < 1% FDR. In light of the high specificity of trypsin [60], it is likely that peptides with either one or no tryptic termini are representative of proteolytic events. We note that these peptides may also be generated by degradation of fully tryptic peptides due to hydrolysis during sample processing or to in-source decay during instrumental analysis, both of which introduce a potential source of ambiguity. To address this concern, we employed a two step filtering procedure. First, we only considered non- or partially-tryptic peptides that were not contained within a longer *observed *tryptic peptide, which reduced the non- or partially-tryptic peptide candidate list to 1656 peptides. Second, we removed non- or partially-tryptic peptides contained within any other peptide, which further reduced the candidate peptide list for examining proteolytic events to 754 peptides (Additional file 5 Table S6). While conservative, this two step filtering approach ensured we considered biologically generated non- and partially-tryptic peptides rather than experimental or analytical artifacts.

**Figure 9:**
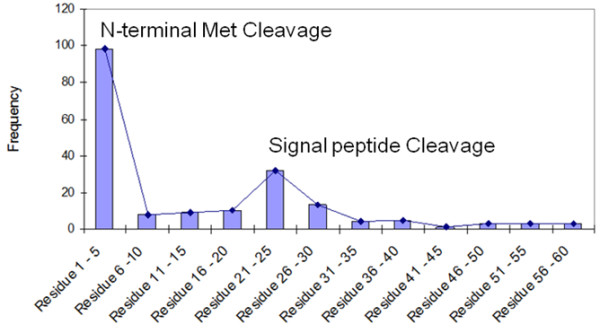
***In vivo *proteolytic cleavage analysis**. Distribution of the curated non-tryptic peptides with residue start positions between 2 and 60 reveals two peaks at residue start positions 1-5 and at residue start positions 21-25 indicative of N-terminal methionine cleavage and cleavage of signal peptides respectively.

A potential drawback of this two-step approach is that identification of proteolytically processed proteins are typically made on the basis of single peptide identifications. However this concern was mitigated by using high resolution and high mass measurement accuracy LC-MS/MS data. If our hypothesis that non- or partially-tryptic peptides likely represent the results of possible proteolytic events were false, we would expect to see a relatively uniform distribution of non- or partially-tryptic peptides across the protein sequence. To the contrary, when the frequency of the occurrence of peptides and the residue start positions between 2 and 60 (Figure 9) are plotted, two pronounced peaks are apparent, one representing residue start positions 1-5 and the other, representing residue start positions 21-25.

**Figure 10:**
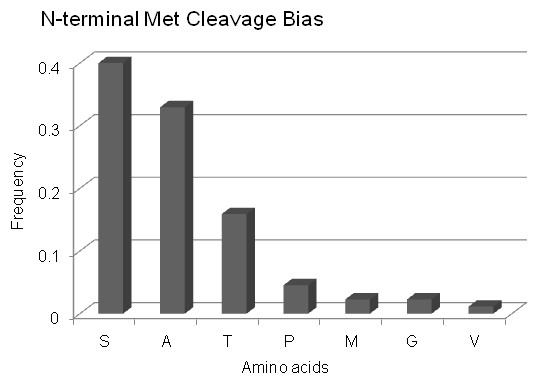
**Experimental annotation of N-terminal methionine cleavage**. The frequency of amino acid occurrence at position P1' reveals a clear preference for small amino acids at P1' in agreement with current N-terminal methionine cleavage rules.

In Figure 9, the peak at residue start positions 1-5, which is comprised almost exclusively (~90%) of peptides with residue start position 2 (see Additional file 5 Table S6) is indicative of N-terminal methionine cleavage, a well-known post-translational modification recognized to be the major source of N-terminal amino acid diversity and thought to play an important role in controlling protein half-life [61,62]. Examination of the peptides with residue start position 2 revealed amino acids with small side chains (Ala, Ser, Gly, Met, Pro, Val, Thr) in the penultimate (P1') position, with Ser (41%) and Ala (33%) most frequently observed (Figure 10), which agrees with established N-terminal methionine cleavage rules; require Met as the P1 residue and amino acids with small side chains as the P1^' ^residue (e.g., Ala, Ser, Gly, Pro, Val, or Thr) [63-67]. Using this set of peptides we confirm cleavage of N-terminal methionine in 88 proteins (Additional file 5 Table S7).

**Figure 11:**
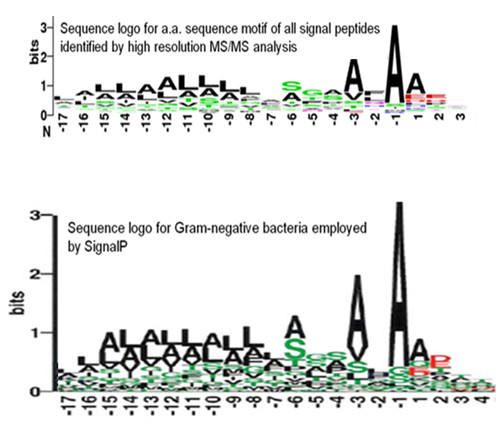
**Experimental annotation of signal peptide cleavage**. The upper panel shows the sequence logo for the amino acid sequence motif of all signal peptides identified by high resolution MS/MS (STable 8). The lower panel shows sequence logo for gram negative bacteria employed by SignalP (Image reproduced from Nielsen et al. 1997).

The peak at residue start positions 21-25 is indicative of signal peptide cleavage, another well-known post-translational modification. Most signal peptides in Gram-negative bacteria range between 20 and 30 amino acids in length, with an estimated average length of 25 amino acids, which is in agreement with our data [68,69]. Examination of the sequence immediately upstream of putative signal peptides (Additional file 5 Table S8; i.e., peptides with residue start positions 20-30) revealed a clear sequence motif [70] (Figure 11) that closely matches motifs used by SignalP [71], shown to be the most accurate in a recent comprehensive assessment of N-terminal signal peptides prediction methods [72]. This provides additional support for using this characteristic set of peptides for identifying signal peptide cleavages. Signal peptides are essential for proper cellular function in both eukaryotes and prokaryotes, targeting proteins for secretion or for transportation to appropriate cellular locations. For example in pathogenic bacteria, secretion of effector molecules is a central hallmark of the host-pathogen interaction in establishing a successful infection. Although algorithms such as SignalP enable genome-based predictions of signal peptides, they almost always lack experimental verification. This lack of verification is of particular concern in the highly sensitive area of recombinant protein production for human therapeutic use where inclusion of amino acids that are actually cleaved in the mature protein might elicit an immune response [73]. Using the current set of peptides we provide genome-scale experimental evidence for signal peptide cleavage for 44 proteins (Additional file 5 Table S8). Analysis of homologs in STM LT2 with > 99% identity by SignalP revealed 41 of these proteins were predicted to possess a signal peptide (Additional file 5 Table S8). Collectively, these results demonstrate the utility of high accuracy MS/MS data for providing label-free high throughput genome-scale experimental confirmation of signal peptide cleavage identifications to improve the quality of the genome annotation. We note that a recent study used a related approach to predict and validate signal peptides in the extracellular proteome of a microbial community [74].

## Conclusions

In this study, we reported the genomic sequence of the bacterial pathogen *Salmonella *Typhimurium (STM) 14028 and demonstrated the use of MS-based proteomics to guide accurate primary genome annotation. Our proteomics data provided experimental confirmation for > 40% of the predicted protein-coding genes, and further improved the genome annotation with 47 start site corrections and the identification of 12 novel genes missed by gene finding programs, importantly these include some that appear to play a role in *Salmonella *pathogenesis.

Comprehensive analysis of post-translational processing events in STM 14028 identified more than 130 signal peptide and N-terminal methionine cleavage events critical for protein function and also revealed a large and complex repertoire of post-translational chemical modifications, including those known to influences various cell processes and several novel chemical modifications.

A major impact of this work on genome annotation efforts is in demonstrating the utility of proteogenomics for high-throughput protein-level experimental annotation, validating and augmenting the *in silico *primary annotation. Even for a well annotated organism like *Salmonella *with a high degree of homology to *E. coli*, we still uncovered a number of annotation errors as well as novel genes. An additional impact of this work is further highlighted in the unique capability of proteogenomics to experimentally annotate *in vivo *post-translational processing events, increasingly recognized to play important roles in prokaryotic biology.

As more distantly related organism are sequenced in efforts to sample more branches of the tree of life, the level of accuracy for current gene prediction programs trained on proteobacteria datasets is expected to markedly decrease, and depending on the GC content of a genome annotation methods suffer accordingly. Thus, the ability of proteogenomics to provide direct protein-level evidence for a significant fraction of predicted protein coding genes will be of significant benefit as a complementary tool in any genome annotation effort. In addition, the data used for proteogenomic annotations may already be generated as part of existing proteomics experiments and then only requires a different analysis method to incorporate the results.

## Methods

### Genome Sequencing

The genome of *Salmonella *Typhimurium strain 14028s was decoded using 454 FLX pyrosequencing technology (Roche) in combination with conventional chain-terminator sequencing at the Genome Center in St. Louis, MO http://genome.wustl.edu. The pyrosequencing efforts resulted in 565,427 reads of 249.3 bases average length, corresponding to 28.8× sequence coverage. These data were complemented with Sanger sequencing reads approximating 4.4× coverage. *De novo *assembly yielded seven contigs (Clifton, unpublished data). A sequence for a different isolate of this strain has recently been deposited at GenBank under accession number CP001363[41]. This sequence is identical to the sequence in this study except for point mutations in 27 loci that await further confirmation. The genome, hence, is a 4.87 Mb circular molecule with a GC content of 52.2%, and contains four presumably functional prophages: Gifsy-1, Gifsy-2, Gifsy-3, and a phage nearly identical to ST64B. Strain 14028s contains a 94 kb plasmid (deposited as CP001362 at GenBank) with over 99% homology to the Typhimurium LT2 virulence plasmid pSLT.

### Automated Annotation

JCVI employs an automated annotation pipeline that identifies genome features in the raw DNA sequence, gathers evidence for function of the features, and assigns functional annotation based on the weight of the evidence.

DNA Feature Identification: Glimmer3 [75] is used to predict protein coding sequences (CDS), tRNAs are identified with the tRNAscan tool [76], rRNA genes and other structural RNAs are identified directly from BLAST [77] matches to Rfam [78], a database of non-coding RNA families.

Evidence for Functional Annotation: JCVI uses a combination of trusted evidence types which provide consistent functional annotation and can be transferred onto genes with high confidence in an automated fashion. The two major trusted evidence types used in the annotation pipeline are:

• *CHAR database: *JCVI's CHAR is a curated database of experimentally verified proteins, source publications, and functional annotations. Each protein entry has detailed annotation including function, gene symbol, and GO terms and evidence codes

• *Trusted Protein Families: *These families currently include JCVI's TIGRFAM protein family models [79] and Pfams [80], both built on Hidden Markov Models (HMMs) as well as NCBI's PRK clusters [81]).

Supporting Evidence for the annotation pipeline includes:

• *BLAST searches against PANDA: *PANDA is JCVI's internal repository of non-redundant and non-identical protein and nucleotide data pulled from public databases that include the latest assembly and protein sequences (e.g., GenBank, RefSeq, UniProt, Protein Data Bank).

• *Computationally derived assertions: *Computations integral to the pipeline include derived physical and chemical metrics including lipoprotein signals (LP) and transmembrane helices (TmHMM, [82]).

AutoAnnotate: AutoAnnotate weighs the evidence from a precedence-ordered list of evidence types-the CHAR database, trusted protein families, best protein BLAST matches from PANDA, and computationally derived assertions-to annotate each protein by assigning, where possible, a function, gene symbol, EC numbers, JCVI functional role category, and GO terms. AutoAnnotate and the databases on which AutoAnnotate runs are freely available for download and installation via the open source repository SourceForge https://sourceforge.net/projects/prokfunautoanno/.

### LC-MS/MS and data analysis

*Salmonella *Typhimurium strain 14028 was grown under four *in vitro *conditions: Luria-Bertani (LB) logarithmic and LB stationary phases, and two acidic minimal media conditions (AMM1 and AMM2) as previously described [21,22]. Given its high osmolarity and nutrient-rich condition, LB broth partially reproduces the small intestine lumen environment, while AMM, providing a low pH, low magnesium, and nutrient-deficient condition, partially mimics the intracellular milieu within the *Salmonella*-containing vacuole (SCV).

The materials and methods used to prepare the protein samples for LC-MS/MS have already been described in full for similar samples [21,23]. Briefly, each sample was lysed, extracted into global, soluble, and insoluble fractions then trypsinized; and further fractionated by ion exchange chromatography. Trypsinized protein samples i.e. peptides were analyzed by ultra high pressure reversed-phase HPLC coupled online to a Thermo Finnigan LTQ ion trap or hybrid LTQ-Orbitrap mass spectrometer in a data-dependent MS/MS mode.

To experimentally validate gene annotations low-resolution MS/MS spectra were analyzed using SEQUEST [13] to search against all possible stop-codon to stop-codon open reading frames (ORFs) ≥ 50 amino acids in length in the STM14028 genome. All identified tryptic and partially tryptic peptides, greater than six amino acids in length, were first filtered by charge state-dependent cross correlation cut-off (Xcorr) scores as follows: a minimum cross-correlation cut-off (*Xcorr*) of either 1.9, 2.2, or 3.3 for 1+, 2+, or 3+ charge states, respectively; and further filtered using a relatively high confidence PeptideProphet [83] cut-off score of 0.9. Partially tryptic peptides were additionally filtered by charge state-dependent cross correlation cut-off (Xcorr) scores as follows: a minimum cross-correlation cut-off (*Xcorr*) of either 3.1, 3.8, or 4.5 for 1+, 2+, or 3+ charge states, respectively. FDR estimated via the decoy database method was < 1% for peptides.

To accurately annotate *in vivo *proteolytic cleavage events high resolution LC-MS/MS spectra were analyzed using X!Tandem [12] to search against the computationally predicted genes in the STM 14028 genome sequence. All identified peptides greater than six amino acids in length, were required to have a Log10 E-value < = -1.3, which corresponds to a 5% probability that the peptide sequence identified as the best hit from the X!Tandem process arose from a random match to a sequence. FDR estimated via the decoy database method was < 1% at the unique peptide level.

To accurately annotate post-translational chemical modifications (PTCMs) high precision MS/MS spectra were analyzed as described below.

Raw data and collated peptide identification information are available to the community as supplemental data at http://omics.pnl.gov/view/publication_1039.html

### USTags and Unrestricted Post-translational Chemical Modifications (PTCM) Search

Data analysis and search for PTCMs were performed using a high resolution LC-Orbitrap FT MS/MS dataset. Data were deisotoped using Decon2LS [84], which implements the THRASH algorithm to determine neutral monoisotopic masses of observed molecular species [85]. Note, Decon2LS is publically available at omics.pnl.gov [86]. The UStags process used for inferring peptide sequences and its PTM mass shifts was described and discussed elsewhere [18,19]. Briefly, de-novo sequences were generated utilizing in-house developed recursive function using neutral monoisotopic fragment masses and sequencing precision of 0.005 a.m.u. and allowing no gaps in sequence tag. Sequence tags are matched in forward and reverse direction against computationally predicted proteins in the STM 14028 genome sequence. If sequence was found to be unique in predicted proteome it was declared a UStag and selected for more precise description from the precursor -fragment spectra pair. A UStag was first aligned within the protein sequence using observed precursor and first fragment mass, preferring tryptic cleavages where possible. If predicted theoretical masses matched observed masses within tolerance of 10 ppm peptide was considered to be non-modified and corresponding spectra explained. The cases where potentially more than one peptide was fragmented were not considered. The remaining spectra containing UStags were subjected for further processing to explain mass shifts with known PTMs. PTM searches are based on the USTag method which provides "near zero" FDR for the tryptic peptide identifications based on decoy database searches. We cannot claim zero FDR since palindromic sequences and sequencing gaps (like Gly-Gly-> Asn or Gly-Ala-> Gln) could occasionally produce false positive hits which are detectable from database inspection. Thus, using the decoy search method of determining FDR this "near zero" FDR would be propagated to modified peptides since the reverse sequence would not produce USTags except in rare cases described above. Thus the FDR of PTM analysis was estimated to be < 1%.

A list of 450 possible PTMs including SNP substitutions as used in this study was obtained from UNIMOD http://www.unimod.org with differential labels and unlikely chemical artifacts manually filtered out. All single and combinations of any two modifications from the list were explored together with the reported residual and terminus specificity of each PTM to produce all plausible explanations of mass shift from non-aligned prefix and suffix sequences with the mass tolerance of 10 ppm. It is important to emphasize that explanation for prefix and suffix mass shifts are completely independent from each other; therefore some modifications could be sufficiently explained even if the whole peptide is not assigned. Expected peptide monoisotopic mass was matched with observed precursor mass from the parent spectra within the 10 ppm. Making the clear numeric cut between two different modification options in an automated fashion is challenging by itself and a decision tree was implemented. Various heuristics and parsimony rules have been historically used to reduce mass ambiguities in order to characterize and estimate counts of modification sites. We argue that in the realm of high resolution mass spectrometry no plausible hypothesis should be rejected except by the strength of experimental evidence. Therefore we developed robust peptide identification scoring function in attempt to extract identification hypotheses which explain UStag containing spectra using the most evidence from observed fragments. A detailed description of the scoring function and default settings used in this study are provided in Additional file 2.

### Informatics and Visualization

Visualization of alignment of detected peptides with the six-frame translation of the genome and the called ORFs was performed using Artemis [87] and/or CLC Genomics Workbench (CLC bio Inc.). BlastP [77] searches of detected peptides corresponding to novel ORFs against the nr database was performed to assign a putative annotation to novel ORFs discovered.

## Authors' contributions

CA, MM, FH, SNP and JNA designed research. CA and HY performed experiments. SP and MM contributed to genome sequencing. MJ, SR, PV, and SNP contributed to *in silico *annotation. CA, SOP, NT, SHP, JLM, MCB, MEM contributed to proteogenomic analysis. SOP, SNP, FH, and MM participated in manuscript preparation. CA, NT, SP, MJ, and JNA wrote the manuscript. JNA and RDS contributed partial funding. All authors read and approved the final manuscript.

## Supplementary Material

Additional file 1 **Tables S1-S4**. Table S1: List of predicted protein-coding genes. Table S2: List of protein-coding genes confirmed at protein level. Table S3: List of genes requiring start site correction. Table S4: List of novel genes. Click here for file

Additional file 2 **Supplemental Text**: A description of the Peptide Identification Scoring Function for High Resolution LC-MS/MS Spectra. Click here for file

Additional file 3 **Table S5**. Table S5: Summary of post-translational chemical modifications observed. Click here for file

Additional file 4 **Figure S1: Selected methylations identified in *Salmonella *of biological relevance with previously observed correlations in *E. coli***. Analysis of the literature identifies modifications in related prokaryotes that have been shown to be biologically relevant and therefore appear to be conserved across *Salmonella *and related organisms. Click here for file

Additional file 5 **Tables S6-S8**. Table S6: Summary of peptides with one or no tryptic ends. Table S7: List of proteins with N-terminal Methionine cleavage. Table S8: List of proteins with Signal peptide cleavage. Click here for file
