# Supplementary material for: Experimental annotation of post-translational features and translated coding regions in the pathogen Salmonella Typhimurium
Source: BMC Genomics. 2011 Aug 25;12:433. doi: 10.1186/1471-2164-12-433 (PMC3174948; doi:10.1186/1471-2164-12-433)
Supplement: Additional file 2 — Supplemental Text: A description of the Peptide Identification Scoring Function for High Resolution LC-MS/MS Spectra. [file 1471-2164-12-433-S2.DOCX]

**Supplemental Text**

**Peptide Identification Scoring Function for High Resolution MS/MS Spectra**

Simple but robust scoring function for evaluation of strength of evidence of peptide (modified or not) identified in high-resolution (60K at 400 m/z used in this study) MS/MS spectra is based on count of predicted fragments matched with the neutral monoisotopic masses inferred from the spectral evidence. Although the function is developed on the basis of USTags de-novo sequencing method and deisotoping accomplished with Decon2ls software it would work with any candidate peptide sequence and list of isotopicaly and charge state resolved masses from the MS/MS spectra.

**P**eptide **Id**entification **S**core (**PIdS**) calculation:

1. Compile theoretical fragments for candidate peptide sequence S of length L_S_
2. Match fragment masses with observed masses within specified tolerance (20 ppm)
3. Construct longest sequence of consecutive matched fragments*
4. Calculate median(MED) relative mass error (ppm) ** and mass error variation (VAR) for compiled sequence (min VAR used in this study is 2ppm)
5. Count forward (NC) and reverse (CN) fragments with mass error within [MED-VAR,MED+VAR]***
6. Count complementary(precursor mass locking) fragments(PML)

$$PIdS=\frac{NC+CN+PML}{L_{S}*VAR}$$

*use b & y fragments for cid & hcd spectra and c & z fragments for etd spectra

**sign is important here so always use mass difference (m_theoretical_ - m_experimental_ )

***use b & y fragments for cid & hcd spectra and c & z fragments for etd spectra; optionally use water and ammonia loss ions(used in this study)

NOTES:

Utility of this function is proven with mass measurement performance comparable or better with those obtained from LTQ_Orbitrap instruments. We have successfully used it with multiple LTQ_Orbitrap, LTQ_Orbitrap Velos and Bruker FTICR instruments. Improvements are possible for this function including evaluation of precursor evidence strength and utilization of isotopic distribution abundance information. As already mentioned this function was developed for de-novo sequencing from hi-res MS/MS spectra and some heuristic regarding charge states of consecutive fragments is already implemented in those procedures. For general peptide identification this logic could be beneficially added to the scoring function.

We observed slight but consistent mismatch between average mass errors in parent and fragments spectra (usually less than 2 ppm). This difference should be accounted for when designing scoring function for precursor confidence.
